# Cognitive Load in Virtual Reality Anatomy Education: Comparing 2D and 3D Learning Experiences

**DOI:** 10.1007/s40670-026-02655-1

**Published:** 2026-02-18

**Authors:** Brandon Lowry, Samantha McGrath, Chad Eitel, Kenneth Ivie, Carolyn Meyer, Becky Wiltgen, Heather Hall, Landon Williams, Samantha Scherner, Tod Clapp

**Affiliations:** Department of Biomedical Sciences, Colorado State University, Fort Collins, CO USA

**Keywords:** Virtual reality, Cognitive load, Anatomy education, Instructional design, Learning outcomes, 3D visualization, Biometric measurement

## Abstract

With virtual reality (VR) becoming increasingly integrated into educational settings, it remains crucial to understand how different formats of educational content impact cognitive processing. This study examined the impact of 2D versus 3D VR modules on cognitive load and learning outcomes in undergraduate anatomy education. Using real-time biometric measurements collected through VR head-mounted displays, cognitive load was assessed as students observed recorded laryngeal anatomy modules presented by virtual instruction utilizing 2D images or 3D models. Despite no significant impact on learning outcomes, participants in the 2D group consistently demonstrated a higher mean cognitive load (M = 0.58, SD = 0.05) compared to the 3D group (M = 0.49, SD = 0.07), indicating a significant reduction in cognitive effort with 3D visualization. Pre- and post-survey results revealed a mixed landscape in perceptions of VR technology, with some students reporting positive views and comfort, while others expressed significant reservations. Notably, however, no significant difference was found between individually reported attitudes and cognitive load, suggesting that students’ cognitive load during the intervention was independent of these variables. Further, prior experience with VR, video game use, and general attitudes toward technology did not significantly influence cognitive load, suggesting that instructional design had a more substantial impact than user familiarity. These findings support the pedagogical value of incorporating 3D VR in anatomy curricula and highlighted the importance of evidence-based design to improve learning outcomes while managing cognitive demand.

**Supplementary Information:**

The online version contains supplementary material available at 10.1007/s40670-026-02655-1.

## Introduction

### Changes in Anatomy Education

Historically, anatomy educators have situated time-intensive, cadaveric dissection alongside lecture-based instruction as the ideal approach for teaching. Recent medical and university curricula changes have reduced the time allocated to the foundational subject by incorporating less time-intensive and more integrated methods, such as problem-based learning [1] and team-based approaches [2].

Compounding the impact of these changes is the noticeable decrease in support for willed body programs, which are essential for traditional dissection-based teaching [3]. Despite the value and impact of these donors on students' education, growing ethical concerns regarding body donation and the increased demand for donated bodies from other disciplines have resulted in a notable negative shift in public opinion [4–6]. Educators have raised concerns over the reduced opportunity for hands-on dissection and observed shortages of donated bodies, with many questioning the level of anatomy knowledge students can reasonably obtain [7].

New technologies, such as virtual reality (VR), have supplemented traditional methods in many curricula. As a computer generated, headset delivered, immersive environment that allows for user interaction, VR offers a scalable and detailed anatomical model that students can manipulate. VR has supported many educators in preserving the quality of anatomy education despite the limitations of curricular changes, dwindling donor programs, and even the logistical challenges during the COVID-19 pandemic [8].

### Adoption of VR Technology

Virtual reality (VR) presents complex anatomical structures in ways traditional methods cannot, as VR can explore three-dimensional models of human anatomy by facilitating intuitive understanding of spatial relationships [9]. Pottle [10] demonstrated VR's effectiveness in anatomy education, noting increased student engagement and improved learning outcomes [10]. Many educators observe positive results with VR, highlighting the capacity to boost engagement and motivation while achieving learning outcomes comparable to traditional methods [11, 12]. Despite this, the adoption of VR in anatomy education has yet to generate a consensus regarding its utility.

Some researchers have cited the higher initial investment and reoccurring costs associated with installing and maintaining VR technologies as notable considerations for both educators and researchers [13–15]. However, research suggests that long-term, maintaining VR technologies are more cost effective, enabling opportunities for large scale deployments that better support educational settings with limited resources [15, 16].

Further compounding the ambiguity associated with the impact of VR-based anatomy education, Tene et al. [17] conducted a systematic review of the technology, revealing a range of opinions on its effectiveness [17]. The ongoing debate highlights the need for further research to identify and understand optimal conditions for VR that most effectively contribute to anatomy education. More specifically, we need to understand how to overcome some of the challenges with individuals using the technology.

### Challenges with VR

A challenge when incorporating VR-based education is the potential for cognitive overload. This can hinder learning outcomes by overburdening students’ working memory [18]. Cognitive overload occurs when the demands on a student's cognitive system exceed their ability to process information, making it difficult for students to manage the complexity of a topic. Cognitive Load Theory (CLT) provides a useful framework for examining the relationship between design and cognitive load. Sweller et al. [19] describe cognitive load as the interaction between a student's cognitive ability and the learning environment [19]. CLT identifies three distinct subtypes of cognitive load: intrinsic, extraneous, and germane. Intrinsic load relates to the complexity or interactivity of the subject being studied. It is specific to the content itself and cannot be altered by the instructional design (i.e., calculus is calculus; neuroanatomy is neuroanatomy) [19]. Teaching methods can reduce the intrinsic load by dividing complex information into smaller, more manageable segments. Extraneous load comes from poor instructional design and may detract from learning goals [19]. CLT theorists recommend that instructional designers reduce extraneous load by delivering content with clear and concise language and learning materials, avoiding unnecessary complexity and distractions. Lastly, germane load pertains to the processes that facilitate learning (e.g., schema construction, automation) [19]. Instructional design choices that enable active learning and engagement can enhance germane load, leading to better understanding and longer-lasting material retention [19].

In VR, factors such as the immersive environment, the volume of information presented, and the level of interactivity can contribute to cognitive overload, as shown by Makransky and Lilleholt [20]. By identifying elements of the VR experience that negatively impact a student's working memory, educators and VR designers can create more effective and manageable learning experiences that mitigate the risk of cognitive overload (i.e., best practices for instructional design in VR).

Addressing the obstacles of VR-based anatomy education is crucial for successful integration of the technology in educational settings. Educators and instructional designers must understand how VR-based education impacts cognitive processes and learning experiences. By identifying best practices for VR instructional design that support the optimal learning conditions, researchers can make design choices that mitigate the adverse effects of VR while maximizing utility and accessibility.

This current study addresses the gap in the literature by examining the interaction of 2D and 3D data, student experience, and student characteristics with cognitive load. The purpose of this study was to evaluate the effect of different variables on student cognitive load and experience. Specifically, researchers sought to:

1. Explore differences in students’ cognitive load when viewing 2D or 3D data in immersive virtual reality.
2. Examine how student characteristics impact cognitive load.
3. Determine the impact of visuospatial ability on student cognitive load.
4. Evaluate the impact of cognitive load on learning outcomes.

## Materials and Methods

### Participants

Seventy undergraduate students in the spring of 2024 were recruited for the primary analyses (2D group *n* = 33, 3D group *n *= 37). Preliminary data were collected across the 2023 calendar year (i.e., spring, summer, and fall 2023 semesters) and modifications in procedure and assessment helped to inform the current procedures. The 2023 data are treated as preliminary due to differences in procedure, issues with data contamination, and edits to assessment questions throughout 2023.

Participants were excluded if they were under 18 years old or not enrolled in an upper-level Human Gross Anatomy course. They received course credit for participation; extra credit and monetary incentives were not offered. After completing the assignments, students were given the opportunity to provide informed consent on whether their data could (or could not) be used in research analysis. Participants with data collection represented in the results are from the 2024 cohort. General demographics were collected, but specific demographics were not paired with experimental and observation data, as many marginalized identities (e.g., female, BIPOC [Black, Indigenous, People of Color]) were not adequately represented within the research team. Additionally, analysis related to cognitive load differences among demographic groups was not conducted on these groups to maintain anonymity of BIPOC students. The program has a small number of BIPOC students which would allow for identification. This study adhered to CSU Institutional Review Board policies and was approved under IRB #4132.

### Study Design

The research team used the mental rotation test scores to randomize participants into two homogeneous groups based on visuospatial ability. Students were divided into a group that participated in either the two-dimensional module or the three-dimensional module (2D *n* = 33; 3D *n* = 37). Before the start of the intervention, all participants completed a pre-test and a pre-survey to measure existing understanding of laryngeal anatomy. These pre assessments also captured student attitude and perception of VR in anatomy education, their prior use of VR and video games, and their overall physical comfort with using VR. Participants then watched a module covering the anatomy of the larynx using either 2D or 3D presentation depending on their experimental group. Both modules were viewed while in immersive VR. A post-test and post-survey were administered immediately following the viewing experience.

### Modules

**Fig. 1:**
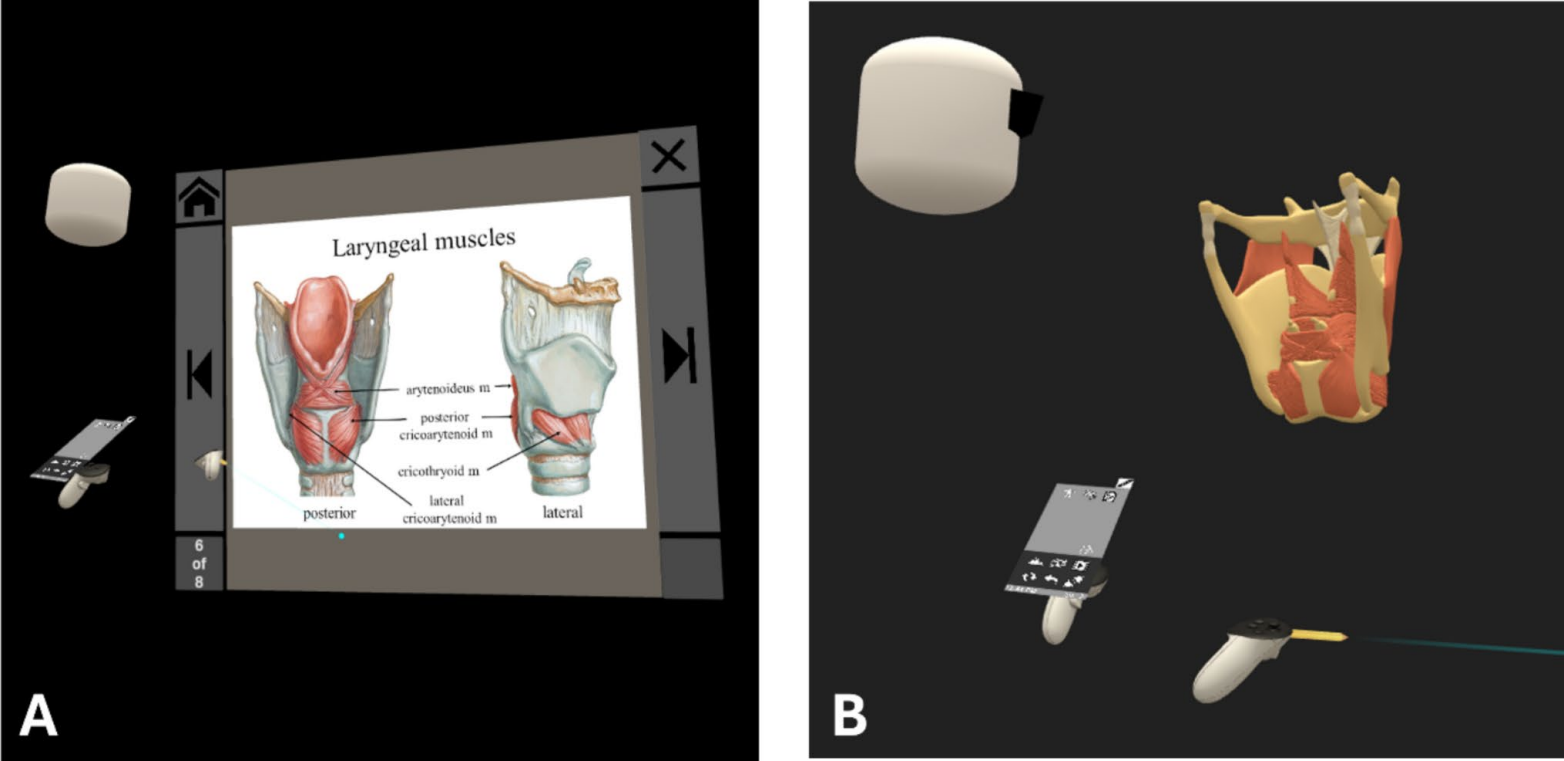
Participants’ view of analogous content in both the 2D and 3D modules. (**A**) In the 2D group, students saw the instructor navigate through a slide show with labeled images of the larynx. (**B**) In the 3D group, students viewed the instructor interact with a dynamic virtual model of the larynx; as the instructor interacted with the virtual model, text labels would appear naming the respective structure (not pictured here). In both images, the white avatar head depicts the senior instructor

The investigators utilized existing software (*Perspectus VR*) to build the two modules shown in VR. The 3D module and the 2D module were conducted in immersive VR. In both modules participants were asked to watch a pre-recorded lesson covering the anatomy of the larynx, a subject that was chosen for its structural complexity. Once in the fully immersive virtual environment, no controllers or model manipulation were used after the recording began. To ensure consistency, a senior instructor recorded both modules to maintain a similar outline, content, and verbiage. The duration of the 3D module was two minutes and four seconds, and the 2D module was two minutes and 45 s. Similarly, a concerted effort was made to keep the scripts in each module as close as possible. Keeping the modules to three minutes or less provided adequate time to review the relevant anatomical information, while limiting the risk of cognitive overload. Students were given the opportunity to watch the 3D or 2D module during VR open lab times, but they were not required to complete both modules. Students were tracked via a sign-up sheet and online survey to determine if they had completed at least one module. Some students watched both modules, but the second viewing was completed at a later time, after the post-test and post-survey were administered. Figure 1 shows what the participants saw during a similar moment in both the 2D and 3D modules. Table 1 describes the differences between the two modules.

**Table 1:** Differences between modules

Condition	Length (min:sec)	Number of images	Narration	Interaction
2D	2:47	10	Same	Student stood and watched
3D	2:15	1 image that is volumized	Same	Student stood and watched

Table 1 highlights the differences between the two modules.

### Measuring Cognitive Load (aim 1)

To examine aim 1, a virtual reality head mounted display (HMD) was used in this investigation: the HP G2 Reverb Omnicept edition. This headset contains biometric sensors to track eye movements (eye-tracking), pupil dilation (pupillometry), and heart rate variability (pulse plethysmography) which achieved ~ 79% accuracy in aligning quantitative cognitive load measures with qualitative reports [21]. The Omnicept reports a cognitive load score based on its algorithm and integrated software. This cognitive load score was collected as participants watched their respective module.

### Student Attitude and Experience (aim 2)

To examine aim 2, a pre-survey was used to examine student characteristics and experiences. The survey included questions about student attitudes toward VR in anatomy education, preference for using VR, physical comfort with VR, how frequently they used VR in the past, and if they played video games in the past (Table 2). Students were also asked if they were retaking the course at the time of the research study. Each survey was coded by two researchers using a deductive approach, categorizing responses into groups (e.g., positive, negative, mixed; comfortable, uncomfortable, mixed; likes VR, does not like VR, mixed feelings toward VR). Key-terms were established prior to initial coding. Codes were added and recategorized following initial coding before being used for a final assessment. Student responses that were unclear to both researchers were excluded from analyses. Table 2 demonstrates this process.

**Table 2:** Pre-survey questions and relevant study goals

Variable	Question	Category	Goal	Final data type
Attitude toward VR in education	“What is your opinion of the use of VR in anatomy education?”	Positive, negative, mixed, unclear	How does student attitude toward VR in education affect cognitive load?	Positive = 1 Mixed = 2 Negative = 3
Attitude toward VR in general	“What is your opinion of VR in general?”	Positive, negative, mixed, unclear	How does student attitude toward VR in general affect cognitive load?	Positive = 1 Mixed = 2 Negative = 3
Physical comfort using VR	“How physically comfortable are you with using VR equipment?”	Comfortable, uncomfortable, mixed, unclear	How does students’ physical comfort affect cognitive load?	Comfortable = 1 Mixed = 2 Uncomfortable = 3
Frequency of Prior Use of VR	“How often do you use VR outside of school?”	None, low, moderate	How does students’ familiarity with VR technology affect cognitive load?	None = 1 low = 2 moderate = 3
Frequency of Prior Video Game Use	“Do you play video games?”	Yes, no	How does students’ prior use of video games affect their cognitive load in VR?	Yes = 1 No = 0
Retaking the course	“Are you retaking this course?”	Yes, no	How does prior exposure to content affect cognitive load?	Yes = 1 No = 0

### Student Perception of Cognitive Load (aim 2)

Similarly, to examine how student experience affected cognitive load, we utilized a post-survey to capture student perception of cognitive load. We adapted the NASA task load index into a survey that, for each question, included an open-ended response as well as 10-point Likert scale [22]. This allowed students to quantitatively rate each of the questions and provide comments. Researchers coded the qualitative responses across six factors – physical demand, mental demand, temporal demand, perceived task difficulty, perceived success, and any negative emotions—as low (1), medium (2), or high (3). The NASA task load index is included as Supplemental Item 1.

### Mental Rotation Test (aim 3)

Visuospatial ability was assessed using the Mental Rotation Test (MRT) initially developed by Vandenberg and Kuse in 1978 [23]. The Mental Rotation Test presents participants with a target image next to four comparison images. Participants are tasked with correctly identifying the two rotated versions with the comparison set by marking them with an X. There are twenty-four questions that participants complete after a seven-minute instruction and demonstration period covering the mechanics of the test. Students were given three minutes to complete the first half of the assessment, followed by a two-minute quiet break, and then three minutes to complete the second half of the assessment. The MRT was given to participants prior to the pre-test and pre-survey to assess aim 3. Assessments were scored by two researchers; points were only given if the student identified both correct images. Researchers then used these data to assign students to research groups and to ensure similar visuospatial ability (as measured by the MRT) among the two groups. Researchers also used this data to explore the relationship between visuospatial ability and cognitive load (aim 3).

## Pre-Test and Post-Test (aim 4)

To examine aim 4, researchers developed a pre-test by using a test/retest protocol with a small group of Human Gross Anatomy alums unaffiliated with the experiment. We finalized a seven-item assessment with six multiple choice questions and one true/false question (see Supplemental Item 2). The pre-test and post-test were designed to assess the following learning outcomes.

*Learning outcomes* *:*

1. Identify the basic anatomy and location of the larynx
2. Recall general functions of the larynx
3. Understand how pitch is produced

In addition to indicating their answer on the pre-test, students also rated their perceived level of confidence in their answer. After participants watched their respective module, researchers administered a post-test that measured the same questions as the pre-test. Responses were examined to identify changes in students’ answers from the pre-test, as well as any changes in how students rated the perceived difficulty and confidence in their answers after viewing the module. Students were not made aware of the correct answers after the pre-test or post-test.

Table 3 demonstrates the alignment between the aims and measurements included in this study.

**Table 3:** Summary of alignment between assessments and aims

Aim addressed	Measurement	Statistical analysis
Aim 1	Biometric cognitive load reported by Omnicept	Independent t-test, ANOVA
Aim 2	pre-post Surveys	Frequencies, regression, correlation
Aim 3	Mental Rotation Test	Group comparison (mean)
Aim 4	pre-post Test	Independent t-test, paired t-test, correlation

### Statistical Analysis

Data were analyzed using R-studio to explore relationships between the modules, measured and perceived cognitive load, and learning outcomes. Researchers compared the average cognitive load score across the module for each participant as a continuous measure of their mental effort during the experience. Independent t-tests were used to assess differences in cognitive load between module groups (Table 3). Independent t-tests were also used to assess the relationship between learning outcomes and cognitive load (Table 3). Paired t-tests were used to assess pre and posttest changes. Learning gain scores were computed and analyzed via independent t-tests. Correlation coefficients were used to examine the relationship between visuospatial ability, cognitive load, and measured and perceived cognitive load. Significance was defined as *p* < 0.05. Descriptive statistics were computed and reported.

## Results

### Participant information

Table 4 shows the participant engagement, sample sizes, and notes pertaining to each activity. Participants represented in this chart and in the results presented are from the 2024 cohort. These participants were undergraduate students enrolled in a prosection based human anatomy course. Specific student demographics were not assessed as some demographics may have allowed for participant identification in the sample size.

**Table 4:** Summary of participant engagement and sample sizes

Stage	Description	2D (*n*)	3D (*n*)	Total (*n*)	Notes
Enrollment	Eligible students			100	
Consent	Completed consent			70	All 100 students were given access to the module
Allocation	Randomized by MRT	33	37		
Pre-Assess	Completed pre-survey	33	37		
Intervention & post-asses	Completed pre-test, viewed module, completed post test	32	36		Missing data *n* = 2
Analyzed	Included in final spring 2024	33	37		

### AIM 1: Data Type and Cognitive Load

**Fig. 2:**
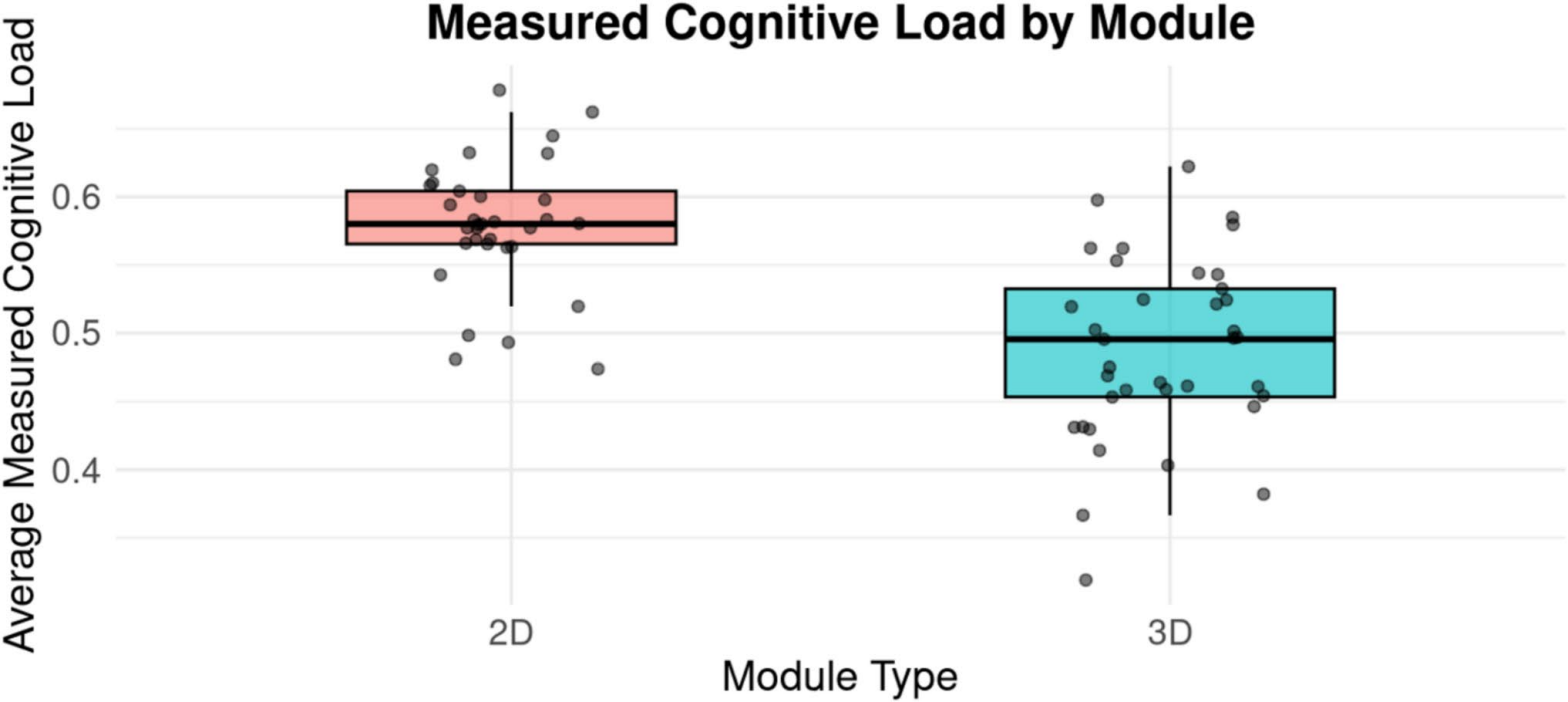
A scatterplot representation of the average cognitive load by module for the individual participants. These data are overlayed onto a boxplot representing group averages

The difference in cognitive load, as recorded by the HP Omnicept, was statistically significant between the 2D and 3D modules. Participants who completed the 3D module displayed a lower cognitive load (M = 0.49, SD = 0.07) than those who completed the 2D module (M = 0.58, SD = 0.05), t(64.86) = 6.63, *p* < 0.001, 95% CI [0.06, 0.012] (Fig. 2). These data suggest that interacting with 3D content in immersive VR reduced mental effort compared to interacting with 2D content. Descriptive values for all measures are summarized in Table 5.

**Table 5:** Summary of descriptive statistics for cognitive load, visuospatial ability, and test performance by module

Measure	2D (*n* = 33) *M* (SD)	3D (*n* = 37) *M* (SD)
Objective Cognitive Load	0.58 (0.05)	0.49 (0.07)
Mental Rotation (MRT)	10.94 (4.12)	11.03 (4.07)
pre-Test Score	5.85 (1.12)	5.69 (0.98)
post-Test Score	5.36 (0.90)	5.11 (0.95)
Perceived Cognitive Load	0.37 (0.16)	0.33 (0.14)

### AIM 2: Student Experience and Cognitive Load

**Fig. 3:**
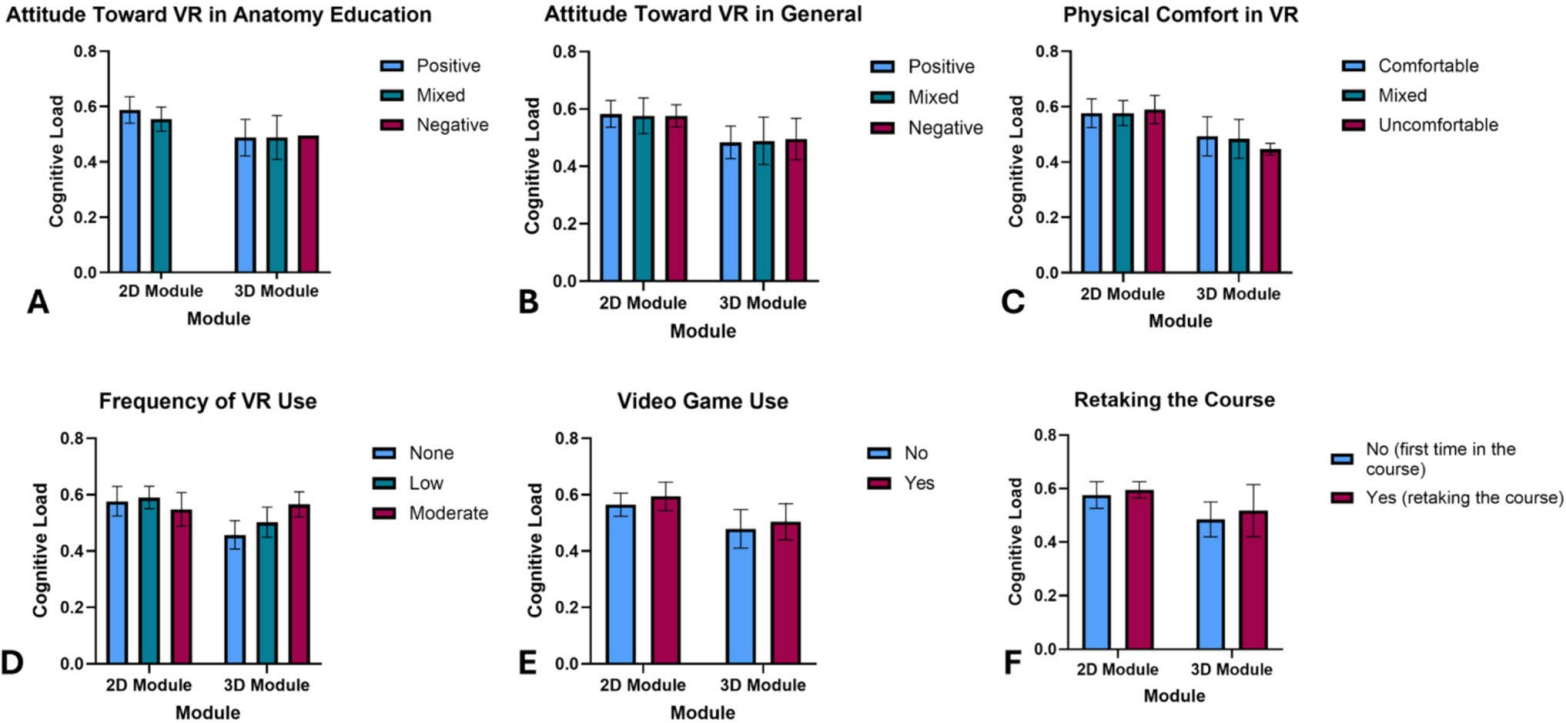
Measured cognitive load based on student responses to the pre-survey. Graphs indicate mean cognitive load ± SD

**Fig. 4:**
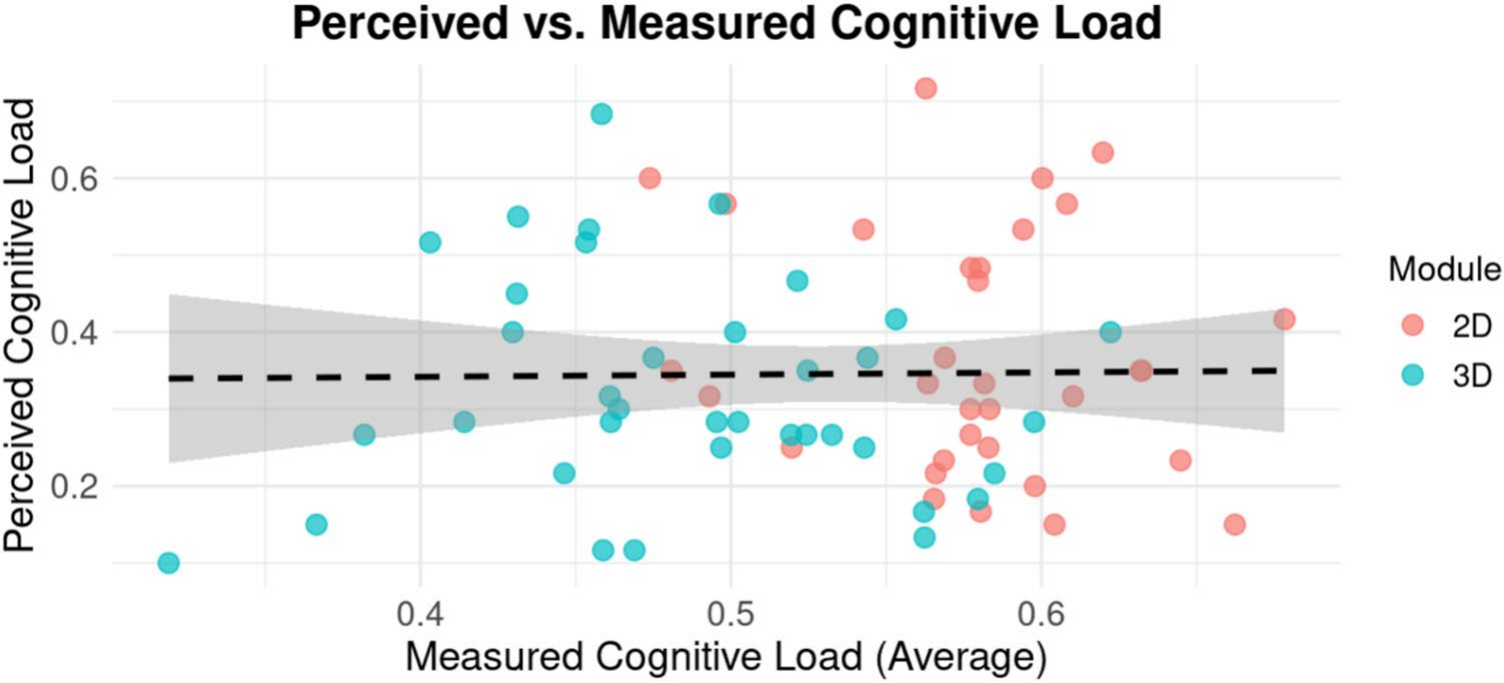
Measured cognitive load and perceived cognitive load by module. Each data point represents the measured and perceived cognitive load for a participant

Student characteristics and experiences as measured by pre and post-surveys (Table 2) did not have a statistically significant effect on measured cognitive load within the 2D or 3D module groups (Fig. 3). The perceived cognitive load from the post-survey was lower for the 3D module (M = 0.32, SD = 0.14) than the 2D module (M = 0.37, SD = 0.16) though these changes were not statistically significant, t(65.07) = 1.29, *p* = 0.20, 95% CI [−0.03, 0.12] (Fig. 4). The measured and perceived cognitive load were not significantly related, r(68)−0.01, *p* = 0.91, which suggests student self-reported cognitive load via the post-survey did not strongly align with their measured cognitive load. Descriptive values are summarized in Table 5.

### AIM 3: Visuospatial Ability and Cognitive Load

The MRT scores did not differ significantly between the 3D (M = 11.03, SD = 4.07) and 2D (M = 10.94, SD = 4.12), t(66.34) = 0.09, *p* = 0.93. This supports the assertion that the randomization process produced comparable visuospatial skills in participants across the two experimental groups. Subsequent analyses demonstrated that MRT scores were not significantly correlated with cognitive load, r(67) = −0.05, *p* = 0.70, which suggests individual spatial ability did not predict cognitive load difference between the two groups. Descriptive values from the MRT are summarized in Table 5.

### AIM 4: Learning Outcomes and Cognitive Load

**Fig. 5:**
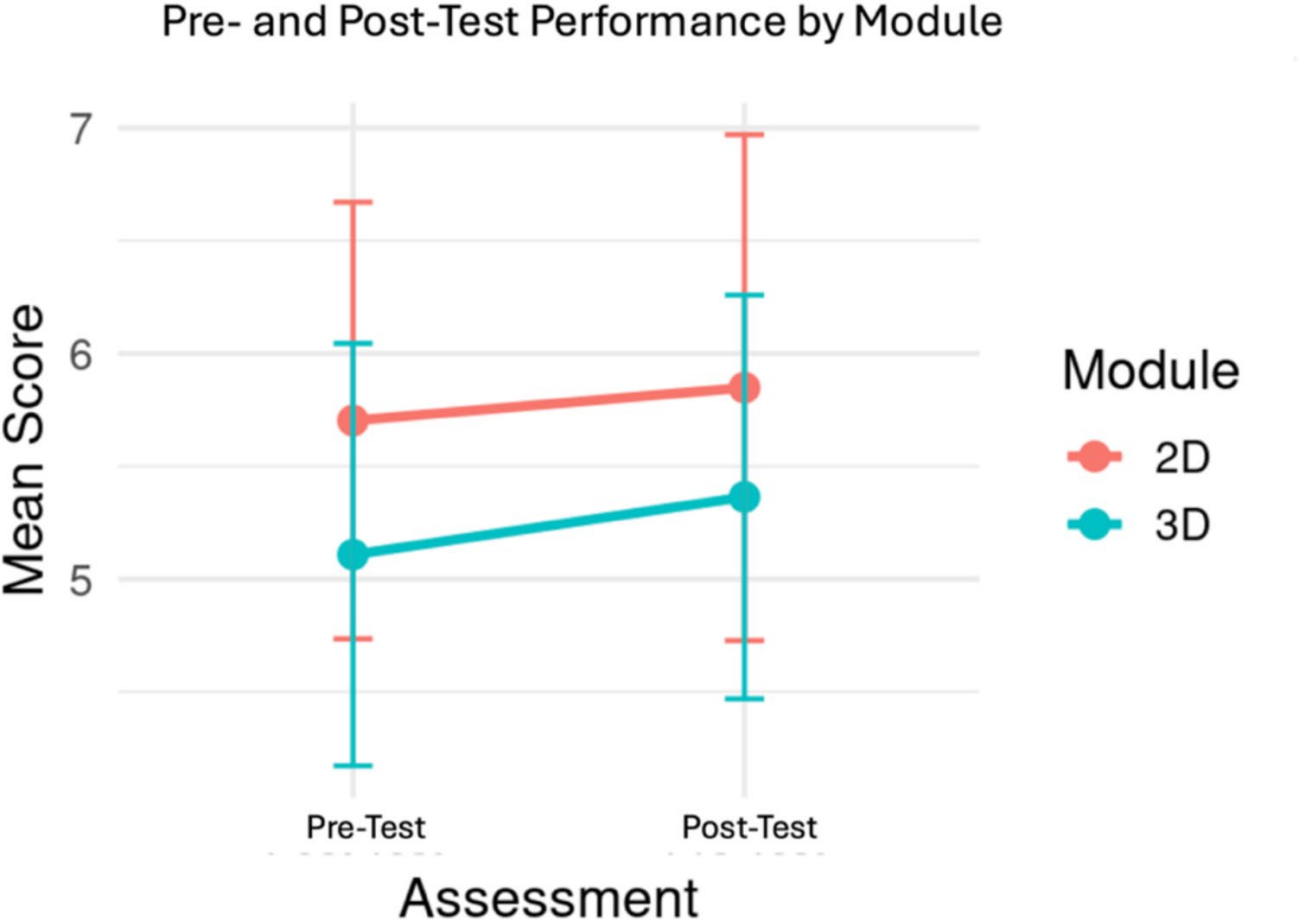
Mean score for pre and post-test performance by module

**Fig. 6:**
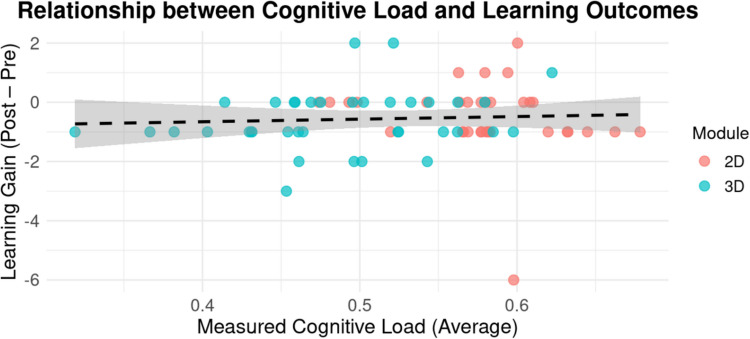
Relationship between cognitive load and learning gains. Each data point represents the learning gain and objective cognitive load for a participant

Analyses within the respective groups revealed significant pre to post-test gains. The 2D group improved at lower level, t(32) = 2.22, *p* < 0.033, than the 3D group, t(36) = 3.57, *p* < 0.001 (Fig. 5). Despite the observed difference, learning gains between the groups did not differ significantly (*p* = 0.69). Additionally, no significant correlation was observed between cognitive load and learning gains within the respective groups (*p* = 0.63), which suggests reduced cognitive load did not directly result in higher scores within a short-term performance context (Fig. 6). Descriptive values for test scores and cognitive load are summarized in Table 5.

## Discussion

This study explored the impact of VR-based education at Colorado State University, assessing cognitive load and learning outcomes in 70 undergraduate students using either a 2D or 3D VR module covering the anatomy of the larynx. Key findings suggest that in VR learning modules, 3D-based content significantly reduced cognitive load compared to 2D-based content. However, the reduction in cognitive load did not result in improved test scores. One explanation is that lower cognitive load may not immediately translate to improved recall, but instead to deeper conceptual understanding in the long-term [20]. Future studies may examine this by including longitudinal study designs and analyses in their research. Student attitude toward VR, as well as their preference and prior use of VR, had little effect on student cognitive load. Additionally, whether a student had previously played video games or taken the class showed little effect on cognitive load across both 2D and 3D interventions. This study provides valuable insight into the impact of data visualization and student characteristics on cognitive load. These results contribute to the understanding of the effectiveness of VR in the educational community and continue the foundation laid by previous works in this field [12].

### Anatomy Education and VR Integration

Recent trends in anatomy education have shown positive effects of VR on student experience and learning outcomes [11, 12, 16, 24–26]. Here, researchers demonstrate that a utility of VR may lie in its ability to lower cognitive load in student learning. The ability of VR to communicate depth and dimension accurately and more intuitively offers an additional resource to traditional learning methods that may improve knowledge gains in the long term [27]. VR not only supports the decrease of time and resources in anatomy education but allows for standardization of education and training [1, 28]. Indeed, as VR becomes increasingly embedded in medical curricula, its potential to shape education continues to evolve [10, 29]. Because discomfort or unfamiliarity can affect engagement, these data suggest a need for strategies to enhance user comfort and address the concerns of those with negative perceptions to broaden effective VR adoption [30]. These suggestions are supported by our findings from aim 2, where we found no significant difference in perceived cognitive load between modules, t(65) = 1.29, *p* = 0.20.

### Cognitive Load

Analysis of cognitive load revealed that students engaging with 3D data in VR experienced lower cognitive load than those using 2D representations (t(64.86) = 6.63, *p* < 0.001, 95% CI [0.06, 0.12]). These findings align with previous work that demonstrated how 3D representations may ease cognitive demands in the absence of measurable improvements in performance [10]. Further, these findings reinforce the importance of instructional design that supports efficient mental processing. When content is presented in a format that aligns with how learners intuitively process information, it appears to reduce unnecessary mental effort and promote more effective learning. Traditional visualization of the larynx in a cadaveric donor is challenging due to the location of the structures of the larynx and the surrounding structures. Students typically struggle with visualizing the larynx and often report learning these structures are challenging. This study showed that presenting an isolated VR 3D anatomical model of the larynx is less cognitively taxing on the learner than static 2D images of the region. However, it is important to consider that the larynx itself as a subject may have contributed to the observed cognitive load in both modules. The 3D visualization of the larynx in VR may have reduced extraneous load by eliminating the need to reconstruct complex 2D images to 3D. Subsequently, more capacity for germane processing may be available (i.e., greater working memory).

In exploring factors that might influence cognitive load, no significant differences were found across student characteristics including attitudes toward VR, prior experience with VR or video games, comfort with the technology, or prior knowledge. Interestingly, moderate VR users did have similar cognitive load between 2 and 3D groups. However, the small sample size for these two groups may have led to the observed cognitive load in these groups. This suggests that the design and presentation of instructional content had a greater impact on cognitive load than individual perceptions and characteristics. As such, educators and designers may benefit from focusing on how information is presented in virtual learning environments over concerns about students’ prior exposure or preferences. While students’ affective responses to VR remain valuable for understanding engagement and overall experience, analysis emphasizes the importance of prioritizing how content is visualized and structured over individual characteristics. In contexts where access to immersive VR may be limited, implementing instructional strategies that incorporate 3D data may still offer significant cognitive benefits. Further, while a direct and immediate improvement on learning outcomes was not observed, there are a few plausible explanations. The sensitivity of the pre and post-test may not have been sufficient, given that it was constructed with a smaller group of students unaffiliated with the study. The limited length of exposure to the respective modules may have also impacted student improvement.

### Experiences Between Modules

While not statistically significant, the post-survey responses indicated that students engaging with the 3D module reported a more positive overall experience compared to those in the 2D group (t(65.07) = 1.29, *p* = 0.20). Interestingly, students in the 2D group were more likely to report feeling hurried or rushed. In the 2D module, students were required to mentally reconstruct spatial relationships to effectively compensate for the lack of depth cues in the static images presented on the 2D panel. This added mental effort may have contributed to the higher cognitive load observed in the 2D group.

In contrast, the 3D module presented information using a virtual model that maintained depth and spatial relationships of anatomical structures, which likely supported a more intuitive and less cognitively demanding engagement with the content. It is important to note that the instructional design differences could have influenced these results, and future research should find ways to create isomorphic protocols where 2D and 3D comparisons are made. Despite the limitation of instructional differences, these findings reinforce the value of immersive, spatially rich design in virtual learning tools—not only for reducing cognitive load, but also for enhancing the overall learner experience.

## Implications for VR-Based Instructional Design

### Educational Practices

Together, these data address our research aim by underscoring how presentation format plays a vital role in learner experience. Our findings with respect to differences in measured cognitive load (*p* < 0.001) advocate for the continued integration of VR into medical anatomy education, emphasizing a need for educational strategies that reduce cognitive load with anatomical content. Reducing mental load through strategic use of spatially intuitive visualizations can help students focus on deeper learning rather than the mechanics of interpreting content. As educational institutions adopt VR across a broader range of learning environments, it will be important to tailor instructional design to meet the evolving needs of learners at various stages. Expanding research into different educational contexts and further understanding the nuances of how different student populations engage with VR can inform the development of teaching strategies that maximize the benefits of immersive learning environments like VR [31]. Good instructional design can optimize intrinsic load and reduce extraneous load for learners.

### Technology Development

From a development standpoint, these results demonstrate the importance of user-friendly, educationally effective tools that are accessible and tailored to educational settings. Emphasis should be placed on content clarity, spatial coherence, and the ability to minimize extraneous load [32]. Incorporating adaptive design features that respond to learner performance and cognitive load in real time may further enhance the effectiveness of virtual instruction. The real-time measurement of cognitive load employed in this study complements existing frameworks for instructional design in virtual reality and underscores the importance of designing VR tools that are not only immersive but also pedagogically aligned.

One consistently observed finding, both subjectively by researchers and directly by the student, was the need for freedom of movement within the physical space. Students who felt agency in moving about the physical environment consistently reported a more positive experience, emphasizing that developers need to consider how the design of both virtual and physical environments may influence not just engagement, but also mental effort. Future studies that examine and manipulate different levels of movement constraints or spatial interactivity within VR will help inform recommendations for static or dynamic movement capabilities, while also clarifying the effect of movement on cognitive load in VR-based environments.

### Limitations

While effort was made to create identical modules, the slight variation between two modules may have introduced some variance in the overall demand placed on the students. Additionally, these results focus on short-term outcomes and the lack of longitudinal analyses limits the generalizability of these findings. The lack of a larger sample due to the progressive nature of the study limits the generalizability of the findings described here. An additional limitation to consider is that this particular study only examined one area of anatomy, the larynx. This could make it difficult to extrapolate the results to other anatomical areas. Although both 2D and 3D formats were presented within the VR system to allow for biometric measurements, the virtual environment is distinct from a typical 2D presentation and this limitation should be considered.

### Future Directions

To expand on the current findings, longitudinal studies are needed to assess how cognitive load and student experience evolve over time with continued exposure to VR. Follow-up research intends to observe a smaller sample size participating in a longitudinal experience. This focus will allow for deeper analysis concerning student cognitive load and how it changes as one becomes more familiar with the technology. This study raised questions about how quickly users adapt to VR and what level of training support is most effective for helping them learn to use the technology. Observing learners across repeated sessions will clarify how familiarity with immersive environments develops and impacts both mental effort and learning outcomes. Additionally, research into the optimal duration and pacing of VR experiences could inform instructional design strategies that balance cognitive demand with engagement and autonomy. Future studies should address these needs with longer-term retention studies, as well as incorporating different levels of interactivity within the experience. As universities launch VR-based curriculum, additional research on curriculum-wide integration will provide valuable insight into the efficacy of VR and, most importantly, rich insight into the relationship between cognitive load and improved learning outcomes.

## Conclusion

This study supports the integration of immersive VR learning in anatomy education by demonstrating that 3D data visualizations reduce cognitive load compared to 2D representations. Continued collaboration among educators, researchers, and developers is paramount in exploring the full potential of VR. Future research exploring additional variables associated with student experience may further contextualize these findings. Refining tools in VR that are both pedagogically effective and accessible across diverse educational settings will further enhance the utility of immersive learning environments in anatomy and beyond.

## Supplementary Information

Below is the link to the electronic supplementary material.

Supplementary file1 (DOCX 23 KB)

Supplementary file2 (DOCX 19 KB)

## Data Availability

The data that supports the findings of this study are available from the corresponding author upon reasonable request.
