# Supplementary material for: Cognitive Load in Virtual Reality Anatomy Education: Comparing 2D and 3D Learning Experiences
Source: Med Sci Educ. 2026 Feb 18;36(2):863–74. doi: 10.1007/s40670-026-02655-1 (PMC13197557; doi:10.1007/s40670-026-02655-1)
Supplement: Supplementary file 1 — Supplementary file1 (DOCX 23 KB) [file 40670_2026_2655_MOESM1_ESM.docx]

The following Supplemental Materials are provided to accompany the submission of “***Cognitive Load in Virtual Reality Anatomy Education: Comparing 2D and 3D Learning Experiences.”*** The materials presented in this document include:

- Supplemental Table 1. Comparison of cognitive load between 2D and 3D modules.
- Supplemental Table 2. Post-survey response frequencies.

##### Supplemental Table 1. Comparison of cognitive load between 2D and 3D modules.

| **Statistics** | **2D Module** | **3D Module** | **t** | **df** | **p-value** | **Mean Difference** | **Std. Error Difference** | **95% CI Lower** | **95% CI Upper** |
| --- | --- | --- | --- | --- | --- | --- | --- | --- | --- |
| Sample Size (N) | 95 | 107 |  |  |  |  |  |  |  |
| Mean Cognitive Load | 0.5749 | 0.5034 |  |  |  |  |  |  |  |
| Standard Deviation | 0.0477 | 0.0571 |  |  |  |  |  |  |  |
| Levene's Test (F) |  |  | 5.221 |  | 0.023 |  |  |  |  |
| t-test for Equality of Means |  |  | 9.587 | 200 | <.001 | 0.071481 | 0.00746 | 0.05678 | 0.08618 |
| Cohen's d |  |  |  |  |  | 1.351 |  | 1.044 | 1.656 |
| Hedges' g |  |  |  |  |  | 1.346 |  | 1.040 | 1.650 |
| Glass's delta |  |  |  |  |  | 1.252 |  | 0.927 | 1.574 |

##### Supplemental Table 2. Post-survey response frequencies.

| **Post-survey Category** | **Group** | **Not Very/Not at All** | **Somewhat/Slightly** | **Demanding/Very Demanding** | **Total Students** |
| --- | --- | --- | --- | --- | --- |
| **Mental Demand** | 2D | 20 | 5 | 6 | 32 |
|  | 3D | 28 | 9 | 2 | 38 |
|  | **Total** | 48 | 14 | 8 | 70 |
| **Physical Demand** | 2D | 29 | 1 | 2 | 32 |
|  | 3D | 34 | 3 | 0 | 38 |
|  | **Total** | 63 | 4 | 2 | 70 |
| **Hurried/Rushed** | 2D | 17 | 6 | 9 | 32 |
|  | 3D | 27 | 8 | 3 | 38 |
|  | **Total** | 58 | 12 | 70 | 70 |
| **Success** | 2D | 2 | 9 | 13 | 24 |
|  | 3D | 2 | 6 | 25 | 33 |
|  | **Total** | 4 | 15 | 38 | 57 |
| **Hard** | 2D | 19 | 7 | 3 | 29 |
|  | 3D | 22 | 11 | 1 | 34 |
|  | **Total** | 41 | 18 | 4 | 63 |
| **IDISA** | 2D | 16 | 7 | 4 | 27 |
|  | 3D | 25 | 8 | 2 | 35 |
|  | **Total** | 41 | 15 | 6 | 62 |
