# Supplementary material for: Cognitive Load in Virtual Reality Anatomy Education: Comparing 2D and 3D Learning Experiences
Source: Med Sci Educ. 2026 Feb 18;36(2):863–74. doi: 10.1007/s40670-026-02655-1 (PMC13197557; doi:10.1007/s40670-026-02655-1)
Supplement: Supplementary file 2 — Supplementary file2 (DOCX 19 KB) [file 40670_2026_2655_MOESM2_ESM.docx]

**Larynx Anatomy: Pre-test**

**same questions used for both pre- and post-test*

**Question 1:**

What is the main function of the larynx?

A) To filter the air we breathe

B) To help you breathe and speak

C) To absorb water from food

D) To regulate blood flow to the throat

How much of a guess is your answer? Circle one:

Completely guessing, partially guessing, not guessing at all

**Question 2:**

Where is the larynx located?

A) Just below the nose

B) In the throat, above the trachea

C) At the top of the lungs

D) In the upper part of the diaphragm

How much of a guess is your answer? Circle one:

Completely guessing, partially guessing, not guessing at all

**Question 3:**

The larynx contains structures known as vocal cords. What do they do?

A) Help in filtering air

B) Help in breathing and producing sounds

C) Aid in the movement of the laryngeal cartilages

D) Control the flow of food and water

How much of a guess is your answer? Circle one:

Completely guessing, partially guessing, not guessing at all

**Question 4:**

True or False: The larynx is also known as the voice box.

A) True

B) False

How much of a guess is your answer? Circle one:

Completely guessing, partially guessing, not guessing at all

**Question 5:**

What happens to the larynx when you swallow food?

A) It contracts to push food down.

B) It moves upward and forward to prevent food from entering the windpipe.

C) It expands to allow more food to pass.

D) It relaxes to facilitate the passage of food to the esophagus.

How much of a guess is your answer? Circle one:

Completely guessing, partially guessing, not guessing at all

**Question 6:**

Which of the following is not a part of the larynx?

A) Thyroid cartilage

B) Cricoid cartilage

C) Arytenoid cartilage

D) Maxillary cartilage

How much of a guess is your answer? Circle one:

Completely guessing, partially guessing, not guessing at all

**Question 7:**

The cricothyroid muscle plays an important role in:

A) Opening the rima glottidis for easier breathing

B) Closing the rima glottidis to protect the airway

C) Increasing the tension of the vocal cords, thereby changing pitch

D) Decreasing the tension of the vocal cords to produce lower sounds

How much of a guess is your answer? Circle one:

Completely guessing, partially guessing, not guessing at all

**Larynx Anatomy: Post-test**

**same questions used for both pre- and post-test*

**Question 1:**

What is the main function of the larynx?

A) To filter the air we breathe

B) To help you breathe and speak

C) To absorb water from food

D) To regulate blood flow to the throat

How much of a guess is your answer? Circle one:

Completely guessing, partially guessing, not guessing at all

**Question 2:**

Where is the larynx located?

A) Just below the nose

B) In the throat, above the trachea

C) At the top of the lungs

D) In the upper part of the diaphragm

How much of a guess is your answer? Circle one:

Completely guessing, partially guessing, not guessing at all

**Question 3:**

The larynx contains structures known as vocal cords. What do they do?

A) Help in filtering air

B) Help in breathing and producing sounds

C) Aid in the movement of the laryngeal cartilages

D) Control the flow of food and water

How much of a guess is your answer? Circle one:

Completely guessing, partially guessing, not guessing at all

**Question 4:**

True or False: The larynx is also known as the voice box.

A) True

B) False

How much of a guess is your answer? Circle one:

Completely guessing, partially guessing, not guessing at all

**Question 5:**

What happens to the larynx when you swallow food?

A) It contracts to push food down.

B) It moves upward and forward to prevent food from entering the windpipe.

C) It expands to allow more food to pass.

D) It relaxes to facilitate the passage of food to the esophagus.

How much of a guess is your answer? Circle one:

Completely guessing, partially guessing, not guessing at all

**Question 6:**

Which of the following is not a part of the larynx?

A) Thyroid cartilage

B) Cricoid cartilage

C) Arytenoid cartilage

D) Maxillary cartilage

How much of a guess is your answer? Circle one:

Completely guessing, partially guessing, not guessing at all

**Question 7:**

The cricothyroid muscle plays an important role in:

A) Opening the rima glottidis for easier breathing

B) Closing the rima glottidis to protect the airway

C) Increasing the tension of the vocal cords, thereby changing pitch

D) Decreasing the tension of the vocal cords to produce lower sounds

How much of a guess is your answer? Circle one:

Completely guessing, partially guessing, not guessing at all
